# Assessment of the FasciMol-ELISA in the detection of the trematode *Fasciola hepatica* in field-collected *Galba cubensis*: a novel tool for the malacological survey of fasciolosis transmission

**DOI:** 10.1186/s13071-016-1303-1

**Published:** 2016-01-16

**Authors:** Annia Alba, Antonio A. Vázquez, Jorge Sánchez, Jorge Fraga, Hilda Hernández, Elizabeth Martínez, Ricardo Marcet, Mabel Figueredo, Jorge Sarracent

**Affiliations:** Laboratory of Monoclonal Antibodies and Biological Models, Parasitology Division, Institute of Tropical Medicine “Pedro Kourí”, Havana, Cuba; Laboratory of Malacology, Parasitology Division, Institute of Tropical Medicine “Pedro Kourí”, Havana, Cuba; Laboratory of Molecular Parasitology, Parasitology Division, Institute of Tropical Medicine “Pedro Kourí”, Havana, Cuba

**Keywords:** *Fasciola hepatica*, *Galba cubensis*, Diagnosis, Epidemiology, ELISA, Multiplex PCR, Snail dissection

## Abstract

**Background:**

Fasciolosis is one of the food-borne neglected trematodioses that has reemerged as a human disease while its effects on domestic animal health remains of significant economic consideration. Being snail-borne disease, the accurate and time-saving epidemiological surveillance of the transmission foci where infected lymnaeid snails occur could be essential to effectively focus or redirect control strategies. For this purpose, the first monoclonal antibody-based immunoenzymatic assay to detect *Fasciola hepatica*-infected snails (FasciMol-ELISA) was recently developed and showed a high sensitivity and specificity when tested in an experimental *F. hepatica* – *Galba cubensis* system.

**Methods:**

Here, we surveyed populations of *G. cubensis* occurring in western Cuba for the assessment of the FasciMol-ELISA in determining natural *F. hepatica* infection in this intermediate host*.* A multiplex PCR, previously developed to detect *F. hepatica* in *G. cubensis*, was used for sample classification. Snail dissection method was also employed as screening technique. A *Χ*^2^ test and a Kappa index were calculated to evaluate the positivity and the level of agreement between the FasciMol-ELISA and the snail dissection methods with the multiplex PCR, respectively.

**Results:**

*Galba cubensis* was found in nine out of 12 sampled localities of which four were positive for *F. hepatica* infection as detected by both immunoenzymatic and PCR-based assays. The overall prevalence was higher than the natural infection rates previously reported for Cuban *G. cubensis* (range from 4.1 to 7.42 % depending on the screening method)*.* No significant differences were found between FasciMol-ELISA and multiplex PCR when determining parasite positivity (*Χ*^2^ = 6.283; *P* = 0.0981) whereas an excellent agreement was also noted (Kappa = 0.8224).

**Conclusions:**

Our results demonstrate the importance of malacological surveys in assessing parasite transmission risk and constitute an alert on the need of accurate measures to control fasciolosis in western Cuba. The sensitivity and specificity of the FasciMol-ELISA as well as its time-saving capacity and the easy of performing the determination of a large number of samples, point at this assay as a novel tool suitable for large-scale monitoring of natural snails populations. To our knowledge, this is the first study that explores natural infection by *F. hepatica* in field-occurring lymnaeid snails using an immunoenzymatic assay.

## Background

The trematode *Fasciola hepatica*, also known as the common liver fluke, is transmitted by freshwater snails of the family Lymnaeidae, in which the asexual reproduction takes place. A large variety of mammals including man, acts as definitive hosts after infection by ingesting contaminated plants or drinking water containing metacercariae [1]. This parasite is widely distributed and considered the main causative agent of the reemerging food-borne trematodiasis known as fasciolosis, which is a significant disease with a considerable global burden in human and domestic animal populations [2, 3].

In Cuba, the biggest archipelago of the Caribbean, the epidemiological scenario of fasciolosis presents two different patterns depending on the definitive host. While human fasciolosis is characterised by reduced but repeated outbreaks and few sporadic cases are reported all year round [4, 5], it is accepted that the disease is an important veterinary problem [4, 6, 7]. Warm temperatures and frequent rainfall throughout the year favour the existence of two species of lymnaeid snails, *Galba cubensis* and *Pseudosuccinea columella*, which act as intermediate hosts of *F. hepatica* in Cuba [8, 9] and other regions of the world [10, 11]. The ecological features of *G. cubensis* (amphibious snail with wide tolerance limits) along with a strong compatibility with Cuban isolates of *F. hepatica* favour its role as the main intermediate host for this parasite in Cuba [12, 13] whereas *P. columella* plays only a secondary role as intermediate host of *F. hepatica* in the region [13]. In fact, only a single population of this species has been found naturally infected with the parasite [8].

In a global scenario of fasciolosis reemergence, the high prevalence of *F. hepatica* in Cuban livestock presumes a high risk of human fasciolosis due to the high rates of transmission of the parasite in nature, mainly related to human activities, e.g. cattle management. Therefore, an accurate control of the parasite is mandatory. However, several factors such as the increase of livestock production to fulfil market demands, and the absence of novel effective drugs and vaccines to counteract parasite’s spreading resistance to triclabendazole (treatment of choice), tackle fasciolosis control only through strategies focused on the definitive hosts [3, 14]. Instead, control strategies based on host snails are a feasible way to overcome these difficulties through integral plans that suit best the epidemiological features of each transmission focus [14, 15]. This necessarily involves surveys of snail habitats in risk areas and periodical analysis of the infection status of intermediate host populations by reliable, simple and time-saving procedures. To this end, a novel diagnostic tool, FasciMol-ELISA, designed to detect *F. hepatica*-infected snails has been recently developed in Cuba [16]. This five-step immunoenzymatic assay uses two monoclonal antibodies (Mab) generated in mice that recognise the total extract of *F. hepatica* rediae. The ELISA showed a high sensitivity (100 %) and specificity (≥98 %) when laboratory-reared uninfected and infected *G. cubensis* and *P. columella* were tested [16].

The aim of the present study is to assess the performance of the FasciMol-ELISA in monitoring *G. cubensis* populations occurring in sites at risk for fasciolosis in western Cuba, where high prevalence of infected livestock and several human disease outbreaks have been reported [4]. A multiplex PCR developed to detect *F. hepatica* in *G. cubensis* [17] was used as a reference method for classification of the samples. This DNA-based assay amplifies a specific segment of the second internal transcribed spacer of the parasite rRNA (ITS2) while amplifying simultaneously a conserved region of the *18S* gene of the snail host *G. cubensis.* The microscopy-based technique of snail dissection, which is used routinely in field surveys of lymnaeid snails [18], was also applied.

To our knowledge, this is the first study that uses an immunoenzymatic assay to detect natural infection of snails with helminths and therefore, constitutes a proof of concept to assess the applicability of immunoassays in the surveillance of parasites in their intermediate hosts. Since malacological surveys can provide useful information regarding *F. hepatica* transmission and infection risks, our results are discussed in the context of what could be relevant to fasciolosis control via intermediate hosts.

## Methods

### Malacological survey of lymnaeid snails

Screening of freshwater snail populations was carried out in water bodies of 12 livestock farms from western Cuba, from January to April 2015, in order to identify those sites harbouring *G. cubensis*. Consent was obtained from the owners and authorities of each livestock farm visited to collect samples. Extensive pasturing was recognised as the principal strategy for livestock feeding in all sampled localities. GPS coordinates of the sites sampled were recorded and mapped using MapInfo v. 11.0 [19] to locate *G. cubensis* habitats. Habitats were classified according to their physical features. Details of each locality sampled are given in Table 1.

**Table 1:** Localities sampled in western Cuba and existing definitive hosts species

Locality	Location (GPS)	Definitive host species	Region/Province
V131	22.5948° N; −83.3721° W	Cows	La Palma/Pinar del Rio
V127	22.5682° N; −83.3761° W	Cows	La Palma/Pinar del Rio
V115	22.55865° N; −83.3596° W	Cows	La Palma/Pinar del Rio
V108	22.5589° N; −83.3678° W	Cows	La Palma/Pinar del Rio
V121	22.5429° N; −83.3613° W	Cows	La Palma/Pinar del Rio
V122	22.5448° N; −83.3546° W	Cows	La Palma/Pinar del Rio
V244	22.53211° N; −83.3515° W	Cows	La Palma/Pinar del Rio
V505	22.7312° N; −82.6594° W	Buffaloes	Alquizar/Artemisa
V503	22.73111° N; −82.6593° W	Buffaloes	Alquizar/Artemisa
Baraca	23.0387° N; −82.4739° W	Sheep	La Lisa/Havana
El Chico	23.01668° N; −82.44561° W	Sheep	Boyeros/Havana
Allende	23.07543° N; −82.39033° W	Cows	Boyeros/Havana

Lymnaeid snails were identified in situ following Pointier et al. [20]. Specimens of *G. cubensis* were collected in their habitats using soft forceps and immediately placed in small containers with soaked filter paper to ensure vitality. Collected snails were carried alive to the Laboratory of Malacology of the Institute of Tropical Medicine “Pedro Kourí”. Sites harbouring populations of the lymnaeid species *P. columella* were also registered.

### Sample processing and detection of *G. cubensis* infected with *F. hepatica*

Collected *G. cubensis* were dissected under a stereomicroscope [18] and carefully checked for intramolluscan stages of *F. hepatica* and other trematodes (parasitological microscopy-based examination). Morphological identification of the rediae and cercariae followed Frandsen & Christensen [21], Dimitrov et al. [22] and Rondelaud et al. [23] and the infection status were registered. Thereafter, each individual was divided into two equal portions with a scalpel.

One of the portions was weighed, mixed with phosphate buffered saline at a ratio of 1 mL buffer/100 mg snail tissue, v/w and homogenised with a Potter homogeniser. The extracts were then tested with FasciMol-ELISA as described in Alba et al. [16]. Briefly, snail extract diluted 1/90 in phosphate buffered saline-tween 20 were individually screened in microtiter plates sensitised with 1E4 anti-*F. hepatica* rediae Mab and blocked with BSA 5 %. Plates were washed and the 4G11 anti-*F. hepatica* rediae Mab conjugated to peroxidase was added diluted 1/1000. The reaction was revealed using a mixture of hydrogen peroxide and orthophenilediame.

The remaining portion of each snail was preserved in 95 % ethanol for further DNA extraction and evaluation via multiplex PCR [17]. This method was considered as a reference technique to check the infection status of *G. cubensis* with respect to *F. hepatica*. Tubes containing each portion of the snail were labelled with the same code to further compare the data from the three diagnostic methods used (i.e. snail dissection, FasciMol-ELISA and multiplex PCR).

### Statistical analysis

Overall and per site prevalence of infection of *G. cubensis* populations with *F. hepatica* were assessed with each diagnostic method and calculated as the number of infected *G. cubensis*/ number of snails examined. A *Χ*^2^ test was used to evaluate statistical differences of positivity to *F. hepatica* detected with either snail dissection or FasciMol-ELISA screening methods against the multiplex PCR. Kappa index [24] was calculated at a 95 % confidence interval to estimate the level of agreement between snail dissection and FasciMol-ELISA in relation to multiplex PCR. The qualitative criteria described by Landis & Koch [25] for this index were used: < 0, no agreement; 0–0.2, insignificant; 0.2–0.4, low; 0.4–0.6, moderate; 0.6–0.8, good; 0.8–1, very good or excellent agreement. All statistical tests were performed in EPIDAT v. 3.1 [26] or Statistica v. 8.0 [27] and differences were considered significant when *P* < 0.05.

## Results

**Fig. 1:**
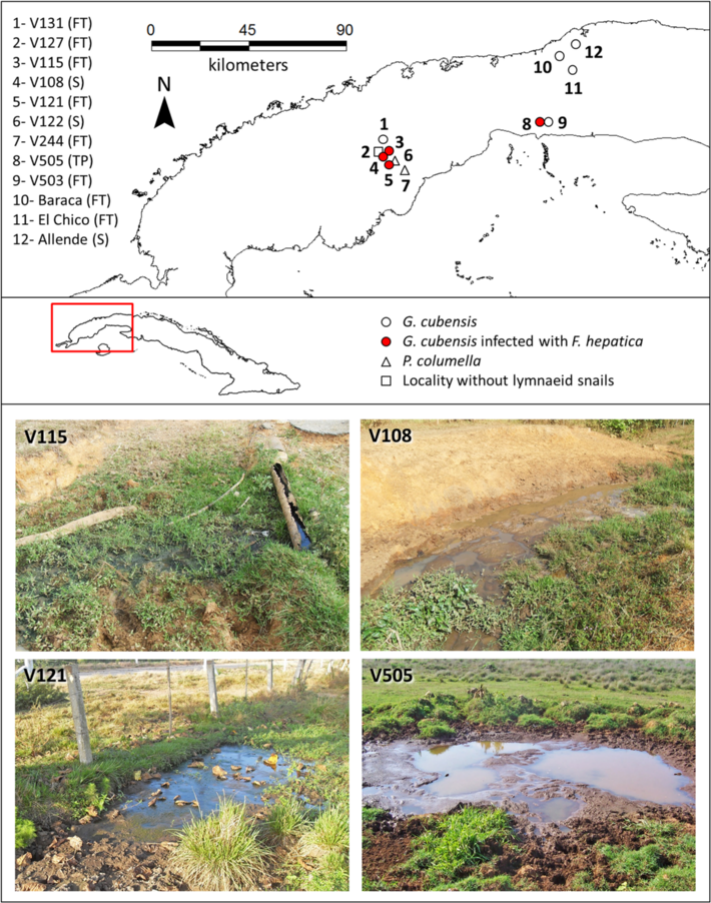
Ocurrence of lymnaeid snails at the localities sampled and positive sites to *F. hepatica*–infected *G. cubensis*. Habitat types are abbreviated as: FT, flooded terrain; S, stream; TP, temporal pond

As a result of the malacological survey, 767 snails identified as *G. cubensis* were collected in nine of the 12 localities visited, mainly in flooded terrains within pasture areas. Only four populations of *G. cubensis* were found infected in the field (Fig. 1). No lymnaeid snails were found in V127 while V122 and V244 only harboured the lymnaeid species *P. columella*.

Table 2 summarises the assessment of the infection status of all field-collected *G. cubensis* by snail dissection, FasciMol-ELISA and multiplex PCR methods. Noteworthy, the band of 450 bp corresponding to *G. cubensis* 18S rDNA, which acts as a positive control of the amplification reaction per sample assayed in the multiplex PCR [17], was absent after the evaluation of 12 samples, most likely due to PCR inhibition. Other studies in which field-collected lymnaeid snails has been screened for infection with *F. hepatica* have also reported PCR inhibition [11, 28] and therefore, these samples were eliminated from the investigation. However, these samples could be assessed by dissection and FasciMol-ELISA; both methods gave negative results. All *F. hepatica*-infected *G. cubensis* detected by dissection were also positive in the FasciMol-ELISA and the multiplex PCR; these corresponded to samples from three sites (V115, V121 and V505). However, snail dissection failed to detect infection in V108, a locality that resulted positive when *G. cubensis* were screened by the other two methods. Noteworthy, no false positive samples were ever recorded. Per site prevalence in positive localities was less than 10 % regardless the method, except for V121 where 20.3 % was registered through snail dissection, 25.8 % with the FasciMol-ELISA and 34.32 % with multiplex PCR.

**Table 2:** Assessment of infections with *Fasciola hepatica* in field-collected *Galba cubensis* through different screening techniques*.* Multiplex PCR was used as the reference technique for parasite detection

Results	Screening method		
	Snail dissection	FasciMol-ELISA	Multiplex PCR
Overall prevalence (%) (no. infected/no. examined)	4.1 (31/755)	5.3 (40/755)	7.42 (56/755)
Positive sites (ID)	3 (V115, V121, V505)	4 (V115, V121, V505, V108)	4 (V115, V121, V505, V108)
*Χ* ^2^	*Χ* ^2^ = 13.69; *P* = 0.0033	*Χ* ^2^ = 6.283; *P* = 0.0981	–
Kappa index (95 % confidence interval)	0.6966 (0.5852–0.8080)	0.8224 (0.7376–0.9071)	–

In addition, a number of snails from V121, V503 and V505 were found harbouring only amphistome cercariae, and several furcocercariae were detected in a snail from Allende by the snail dissection method. Both multiplex PCR and FasciMol-ELISA confirmed the absence of *F. hepatica* in these snails, first observed through dissection, indicating the high specificity of both techniques.

## Discussion

### Digenean infections in malacological surveys

For a digenean to be successfully transmitted, both parasite and host species, must occur in a site where ecological conditions suit parasite’s life-cycle requirements [29]. The distribution of a given parasite in the field is mostly random and linked to that of the intermediate host, which normally has narrower dispersal capacity than definitive host species [29]. Digenean infection in the intermediate host is a probabilistic event [9] but chances may increase if high densities of definitive hosts also occur. Therefore, this study was conducted in livestock farms, some of which have been reported as transmission foci of *F. hepatica* [9].

*Galba cubensis*, the main intermediate host of *F. hepatica* in Cuba, was recorded in nine of 12 sampled localities, a fact probably related with the wide distribution of this species throughout the island and its preference for anthropic ecosystems [12]. Although snails infected with *F. hepatica* were detected in only four localities, a high risk of transmission is presumed in the remaining given the coexistence of susceptible host snails and definitive hosts. Management strategies of livestock to avoid the contact with the snail hosts (e.g. field drainage, fences, pasture rotation) could be applied in the identified *G. cubensis*-positive sites to diminish the risk. The accurate and periodical mapping of intermediate host populations within these localities would allow determining differential risks of *F. hepatica* transmission among particular sites of each locality. In this sense, V122 and V244 face a lower risk of local *F. hepatica* transmission due to the presence of only single lymnaeid species, *P. columella*. In V127 a null risk can be presumed at the moment since no lymnaeid snails were registered.

Recently, the first study on prevalence and intensity of natural infections with *F. hepatica* in *G. cubensis* in Cuba, using snail dissection as screening method, has been reported [9]. Surveys carried out by Vázquez et al. [9] from January to March 2013 were conducted in eight localities of western Cuba, some of which were also investigated in our study (i.e. V108, V505, V122, V127 and V131). Therefore, the cited work could be considered as a baseline to analyse fasciolosis transmission in this region and particularly, in some of these sites and the comparison with our results indicates that V108 and V505 remained as active transmission foci of *F. hepatica*. Conversely, populations of *G. cubensis* from V131 which have been previously reported to harbour *F. hepatica* [9] were negative to parasite infection in the present study. The differences that may appear in transmission sites over time can be related to several factors: (i) transmission may have ceased in the sampled sites due to recent severe droughts; (ii) sampling failed to collect infected snails due to parasite and snail’s ecology (see Vázquez et al. [9]); and (iii) transmission may have ceased due to effective control strategies. However, the latter seems unlikely since anthelminthic treatment of livestock has been the main control measure applied in these localities. Since anti-flukicidal drugs do not prevent reinfection (and continuous treatment is costly), this solely strategy is not sufficient to control the transmission of *F. hepatica*, i.e. if no other management policies related to the animals and/or environmental sanitation are also applied [3, 14]*.*

Our results confirm the role of *G. cubensis* as an important intermediate host of *F. hepatica* in Cuba and also of other trematode species. This lymnaeid snail has been found naturally infected in Cuba with amphistome and furcocercariae identified as members of the families Paramphistomatidae and Schistosomatidae, respectively [9, 30].

### Prevalence of *F. hepatica* in natural populations of *G. cubensis*

The prevalence of infection with *F. hepatica* in natural populations of lymnaeid snails is variable and depends on several factors such as the species of intermediate host, geographic location and time of the year [29]. On the other hand, the screening method used can introduced a significant bias in the assessment of natural prevalence depending on their sensitivity and specificity. However, the overall prevalence of infection with *F. hepatica*, recorded in the present study by either of the screening methods used, does not differ greatly from what has been informed after large-scale monitoring of the infection status of lymnaeid snails. Several investigations aiming to determine natural infections with *F. hepatica* in *G. truncatula* have reported less than 10 % prevalence by microscopy-based techniques [31–33] and PCR-based assays [28, 34]. In addition, previous studies on natural populations of *G. cubensis* in Florida [10] and the western region of Cuba [9] have also reported low prevalence of *F. hepatica* infections in snails of 1.51 % and 2.94 %, respectively*.*

However, even low prevalence levels of *F. hepatica* infections in a particular population of intermediate hosts may pose a high risk of parasite transmission [9]. Taking into account that tens of rediae and hundreds of cercariae can be developed from a single miracidium of *F. hepatica*, a single infected snail can provide enough parasite metacercariae to infect new definitive hosts [1, 23]. Abrous et al. [35] reported averages of 11.2 and 427 for the redial burden and emerging cercariae, respectively, in experimental monomiracidial infection of *G. truncatula* with *F. hepatica.* Moreover, highly compatible parasite - intermediate host combinations can lead to significant increases in parasite prevalence and intensity of infection therefore, increasing the risk of transmission [13, 36]. In this sense, the mean value of *F. hepatica* rediae recorded in naturally-infected *G. cubensis* from the same region of western Cuba (24.21 ± 20.02) is higher [9] than the redial burden informed by Mage et al. [31] in field-collected *G. truncatula* from France (12.3–17.1). Our results alert on the occurrence of several high risk sites of *F. hepatica* transmission and the need to revise current strategies to control animal fasciolosis in the western region of Cuba.

### Detection of *F. hepatica* in snails: applicability of FasciMol-ELISA

Screening methods to detect *F. hepatica* in its intermediate host are the foundations of epidemiological surveillance of parasite transmission. Because infection prevalence in snails is usually low [9, 10, 33], it becomes paramount to survey different freshwater bodies within the risk areas with a subsequent screening of large numbers of snails per site. Therefore, it is essential to count on diagnostic methods to detect *F. hepatica* transmission foci that allow assaying large samples through simple, time-saving and standardised procedures of suitable sensitivity and specificity.

Techniques based on microscopy (e.g. snail dissection or cercarial shedding) have been frequently applied to detect *F. hepatica* in its intermediate hosts [31, 33, 37]. However, these methods may have some drawbacks (see Caron et al. [18] for a more comprehensive overview). Dissection is the most widely used technique to detect *F. hepatica*-infected snails and is suitable to assess prevalence and intensity of infection [9, 31, 33]. However, specificity may be an issue since trematode identification relying only on the morphological features of parasite intramolluscan stages is quite difficult [18]. Besides, sensitivity in early prepatent infections is acknowledged as a major disadvantage of this technique [10, 18]. Even when visualisation of the parasite in its intermediate host is an irrefutable diagnosis, DNA-based assays are superior methods regarding sensitivity and specificity [10, 11, 18]. Therefore, in the present study a multiplex PCR was used to accurately assess the infection status of natural *G. cubensis* with *F. hepatica.*

Table 3 summarises our qualitative interpretation regarding several features of the screening methods used to investigate the infection status of *G. cubensis* that derives from discussing the data obtained and our own experience. We strongly believe that beyond raw numbers, only the global balance of the features of the methods in relation to the pursued goal can lead to the proper selection of a diagnosis technique. The multiplex PCR allowed the detection of a larger number of positive samples while the lowest prevalence was assessed when using snail dissection, a fact directly related with sensitivity. Similar to our results, differences regarding positivity between classical techniques and molecular biology methods in examining field-collected lymnaeid snails have been reported [10, 11]. In fact, Kaplan et al. [10] demonstrated that detection of *F. hepatica* in infected *G. cubensis* using a parasite-specific DNA probe was significantly more sensitive than snail dissection during the first three weeks post-infection. However, it should be taken into consideration that the unsuitability of certain field samples to analysis via multiplex PCR due to the presence of PCR inhibitors, directly impacts the sensitivity of the assay since these snails must always be excluded from the study.

**Table 3:** Qualitative comparison (from 0 to +++) of the techniques used for the detection of *F. hepatica* in *G. cubensis*

	Sensitivity	Specificity	Ease of application	No. samples/unit time
Snail dissection	+	+++	+	+
Multiplex PCR	+++	+++	++	++
FasciMol-ELISA	++	+++	+++	+++

We considered FasciMol-ELISA as second in place regarding sensitivity, but it is noteworthy that no significant differences were observed between the positivity recorded by the multiplex PCR and the immunoenzymatic assay (*P* = 0.0981). In fact, the excellent level of agreement attained between the two methods (Kappa index > 0.8) allowed the detection of *F. hepatica* in snails from four sites. The difference in the number of positive samples between the multiplex PCR and the FasciMol-ELISA is likely related with the time points at which the parasite can be detected by each method*.* The limit for detecting DNA of *F. hepatica* in the multiplex PCR is lower than the DNA amount of a single miracidium enabling the detection of the parasite during all periods of the infection in snails [17]. Instead, the detection time frame of the parasite by the sandwich ELISA is extended only up to 80 % of the duration of the prepatent infection of *F. hepatica* in *G. cubensis*, i.e. starting after the first week post-infection [16]. Moreover, possible bias in the distribution of parasite larvae between the two portions of snails that were independently assayed by each method may have negatively influenced FasciMol-ELISA positivity, especially in early prepatent infections, given its lower analytical sensitivity compared to multiplex PCR.

The three screening methods showed high specificity regarding *F. hepatica* (specific) detection and it is noteworthy that no false positive results were obtained. However, other studies indicated that snail dissection is less specific than DNA-based techniques in detecting infections with *F. hepatica* [10, 11]. This discrepancy could be related to the fact that methods based on microscopy depend greatly on the individual expertise, a variable that is difficult to replicate and standardise when this method is applied in different laboratories.

The easy of performing and interpretation of a diagnosis is another issue that must be taken into account when discussing screening methods and in this sense, the immunoenzymatic assay may be superior (Table 3). The FasciMol-ELISA is a five-step technique that can be performed in a clinical parasitology laboratory without the requirements and facilities needed in molecular biology laboratories. Also, processing of snails for its evaluation in the sandwich ELISA and the interpretation of the result is quite simple (see Alba et al. [16] for details) and tends to favour the easy application of this method. Conversely, specific training and extreme thoroughness are mandatory for snail dissection to overcome low sensibility and specificity issues of this technique. On the other hand, PCR-based assays involve several steps that require careful manipulation in order to avoid possible DNA degradation and contamination [18].

In addition, when assaying a great number of snails these latter difficulties directly entail greater time for diagnosis compared to the sandwich ELISA. Longer time for diagnosis with the multiplex PCR essay is related mostly to DNA extraction (Chelex® extraction is a 5 h procedure [38]) and to the visualisation of the amplified fragments of each sample on agarose gels, whereas dissecting one-snail-at-a-time during large-scale monitoring is a time-consuming and exhausting procedure. Instead, the time-saving capacity of the FasciMol-ELISA to diagnose a large number of samples (i.e. around 200 samples) in only 6–7 h, makes it a suitable procedure to screen field-collected snails during epidemiological surveys.

As mentioned above, the selection of a particular diagnosis technique must ponder its advantages and drawbacks in view of the goal pursued. In the present study, infection with other digenean was noticed only through snail dissection meaning that this is a suitable method to study intermediate host bionomics, as well as to characterise parasite infection (e.g. larval burden, parasite development) [18, 31, 32]. On the other hand, the high sensitivity and specificity of the multiplex PCR endorse its application as an accurate diagnostic tool when a high level of completeness is required, or as reference method in the evaluation of other detection techniques. Its relatively high cost compared to other screening methods is an issue that might be considered during large-scale snail surveys, especially for laboratories with limited financial resources. Significantly, our results demonstrate that FasciMol-ELISA is an appropriate, simple, standardised and time-saving method to investigate the infection status of *G. cubensis* with *F. hepatica* during large-scale surveys. The sandwich ELISA was sensitive and specific enough in determining the transmission foci of the parasite as no positive site was disregarded, no false positive result was attained and a higher positivity was achieved with this method compared with the microscopy-based technique, which is still considered by many authors as the gold standard method (see Caron et al. [18]). The fact that FasciMol-ELISA is an immunoenzymatic assay similar to others already used for diagnosis of fasciolosis in the definitive hosts [4, 39, 40], could possibly help its introduction in clinical parasitology laboratories where the basic requirements (regarding equipment and technical capacities) are created, even in low-income countries. In addition, antibodies’ positive reaction when *F. hepatica-*infected snails are assayed in the FasciMol-ELISA is significant in terms of colour development (see Alba et al. [16]) thus, a presumptive diagnosis can be performed by eye if no spectrophotometer is available.

## Conclusions

The present study reports the existence of several risk sites and transmission foci of *F. hepatica* within a number of farms in western Cuba and alerts about the necessity of a more accurate management of livestock. Our results demonstrate the importance of malacological surveys for supplying the necessary knowledge on the transmission dynamics of this parasite for veterinarians and epidemiologists in order to design effective strategies for fasciolosis control. In this sense, the FasciMol-ELISA proves to be a novel, reliable and suitable tool that could be recommended for examining large numbers of field-collected *G. cubensis.* Moreover, its potential applicability to detect *F. hepatica* in other lymnaeid species and even in definitive hosts [16] might be a useful feature that may extend the use of FasciMol-ELISA as a unique diagnostic method. To our knowledge, no other immunoenzymatic assays have been reported to detect helminth-infected snails, therefore the present study constitute a novel proof of concept on the suitability of immunoassays for epidemiological surveillance of the host snails of *F. hepatica* and digenean parasites.
